# Differentiating Responders and Non-responders to rTMS Treatment for Disorder of Consciousness Using EEG After-Effects

**DOI:** 10.3389/fneur.2020.583268

**Published:** 2020-11-20

**Authors:** Renhong He, Jianzhong Fan, Huijuan Wang, Yuhua Zhong, Jianhua Ma

**Affiliations:** ^1^School of Biomedical Engineering, Southern Medical University, Guangzhou, China; ^2^Department of Rehabilitation Medicine, Nanfang Hospital, Southern Medical University, Guangzhou, China

**Keywords:** repetitive transcranial magnetic stimulation, disorder of consciousness, EEG after-effects, relative power, improved consciousness

## Abstract

**Background:** It is controversial whether repetitive transcranial magnetic stimulation (rTMS) has potential benefits in improving the awareness of patients with disorder of consciousness (DOC). We hypothesized that rTMS could improve consciousness only in DOC patients who have measurable brain responses to rTMS.

**Objective:** In this study, we aimed to investigate the EEG after-effects induced by rTMS in DOC patients and attempted to propose a prediction algorithm to discriminate between DOC patients who would respond to rTMS treatment from those who would not.

**Methods:** Twenty-five DOC patients were enrolled in this study. Over 4 weeks, each patient received 20 sessions of 20 Hz rTMS that was applied over the left dorsolateral prefrontal cortex (DLPFC). For each patient, resting-state EEG was recorded before and immediately after one session of rTMS to assess the neurophysiologic modification induced by rTMS. The coma recovery scale revised (CRS-R) was used to define responders with improved consciousness.

**Results:** Of the 25 DOC patients, 10 patients regained improved consciousness and were classified as responders. The responders were characterized by more preserved alpha power and a significant reduction of delta power induced by rTMS. The analysis of receiver operating characteristic (ROC) curves showed that the algorithm calculated from the relative alpha power and the relative delta power had a high accuracy in identifying DOC patients who were responders.

**Conclusions:** DOC patients who had more preserved alpha power and a significant reduction in the delta band that was induced by rTMS are likely to regain improved consciousness, which provides a tool to identify DOC patients who may benefit in terms of therapeutic consciousness.

## Introduction

Coma commonly occurs as a result of severe brain injury, including traumatic brain injury (TBI), stroke, and hypoxic-ischemic encephalopathy (HIE). Many patients who are in a coma for more than 2 weeks, will emerge into a disorder of consciousness (DOC), which can be classified as either unresponsive wakefulness syndrome (UWS) or a minimally consciousness state (MCS) (1). UWS, previously called the vegetative state, is defined by a patient having periods of preserved behavioral arousal without responsiveness to an external stimulus. MCS is characterized by a minimal, but definite, behavioral sign of conscious awareness and responses to external stimuli in a patient (2). Once a patient is diagnosed with a DOC, a poor prognosis is assumed, and withdrawal of life-sustaining therapies is likely. However, a recent study of DOC patients with a 10-year follow-up showed that a substantial proportion of patients who were unable to follow commands upon inpatient rehabilitation admission achieved independence in cognitive, mobility and self-care functions with improvements evident up to 10 years post-injury. This suggested that individuals with DOC may benefit from ongoing functional monitoring and updated care plans during the post-acute phase (3). Unfortunately, 54% of DOC patients do not receive clinical rehabilitation, which decreases their possibility of recovery (4). In addition, the lack of efficacious treatments for arousal awareness is a critical factor in the limited rehabilitation for DOC patients. Recently though, repetitive transcranial magnetic stimulation (rTMS), a non-invasive brain stimulation (NIBS) technique, has shown promise as a potential approach to improve consciousness in DOC patients.

Consciousness is a multifaceted concept that has two major components, awareness of self and the environment, and wakefulness (5). An essential requirement for consciousness is the brain's capacity to rapidly integrate information across different specialized cortical areas (6) and a disruption in interregional cortical connectivity within the frontal-parietal network is thought to be associated with the loss of consciousness (7). Therefore, it is thought that the recovery from DOC is dependent on the ability of the brain to recover neural circuits and functions through neuronal plasticity, which are involved in conscious behaviors. In addition, rehabilitative interventions can improve functional outcomes by promoting adaptive functional and structural plasticity in the brain, which can be induced by an approved NIBS treatment. Transcranial magnetic stimulation (TMS) is a NIBS technique that can modulate cortical excitability and has shown significant therapeutic promise in treating neurological disorders (8). rTMS is one type of TMS characterized by an output of many pulses that can modulate the cortical activity temporally beyond the stimulation period and spatially beyond the stimulation site (9). Low frequency (≤ 1Hz) and high frequency (≥5Hz) rTMS protocols have a strong dichotomy in that the former can decrease the cortical excitability to form long-term depression (LTD) and the latter can produce facilitating oscillatory activity to form long-term potentiation (LTP) (10). It is known that LTD and LTP were involved in neural plasticity, and correlate with neurological disorders. Koch et al. (11) demonstrated the impairment of LTP-like together with normal LTD-like cortical plasticity in patients with Alzheimer's Disease (AD) and TMS has been introduced as a novel therapeutic approaches in AD (12). In the neural system, the LTP/LTD-like mechanism is thought to play important roles in brain network recovery, and is considered a promising way for improving DOC outcome (13).

In one study, it was claimed that high-frequency rTMS for 5 days over the left primary motor cortex (M1) may improve the awareness and arousal of DOC patients and that EEG may be a potential biomarker for the therapeutic efficacy of 20 Hz rTMS (14). Several lines of evidence have shown that rTMS over a different region, the dorsolateral prefrontal cortex (DLPFC), can alter the excitability of the cerebral cortex and thereby improve the disturbance of consciousness in some UWS patients (15, 16). Conversely, two randomized and sham-controlled studies did not provide evidence that showed therapeutic efficacy of 20 Hz rTMS over the M1 of patients in chronic vegetative states (17, 18). In addition, in a pilot study, a single session of high-frequency rTMS delivered over the right DLPFC did not produce any significant clinical changes at the group level (19). Furthermore, a recently published guideline (20) for rTMS does not make any recommendation for its use in DOC because there has been no high-quality study that demonstrates its efficacy. Taken together, there is no debate in the ability of rTMS to modulate cortical excitability, but there is insufficient evidence to support its therapeutic application in DOC patients.

Recording of bioelectric brain activity using EEG during and after transcranial stimulation enabled quantification of the after-effect of brain responses. We hypothesized that rTMS can improve consciousness in specific DOC patients (i.e., the responders), which are DOC patients who have certain measurable brain responses to rTMS. In addition, in this study, we aimed to differentiate responders and non-responders using the EEG after-effects induced by rTMS and proposed a prediction algorithm to screen for responders to rTMS treatment.

## Materials and Methods

### Patients

Twenty-five patients with DOC were enrolled in this study according to the inclusion and exclusion criteria. The inclusion criteria were as follows: (1) classifying as a vegetative state according to internationally established criteria (21); (2) DOC condition lasting more than 3 months; (3) age ranged between 18 years and 70 years; (4) no use of centrally acting drugs; (5) first-ever brain injury; and (6) no other neurological/psychiatry. The exclusion criteria were as follows: (1) history of epilepsy within 1 month; (2) a pacemaker or other metallic implants in the head; (3) skull defect; (4) serious complications, including heart failure or renal failure. This study was conducted in accordance with the Declaration of Helsinki and approved by the Medical Ethics Committee of Nanfang Hospital (China). All patients and their families were given information regarding the procedures of this study and gave written informed consent.

### Design and Stimulation Procedures

Prior to the rTMS/EEG session, the resting motor threshold (RMT) was measured. Electromyography signals were recorded via disposable surface electrodes placed at the muscle of the left abductor pollicis brevis. According to the recommendations of the International Federation of Clinical Neurophysiology Committee (22), the stimulation intensity was determined based on the RMT which was defined as the minimum output intensity that evoked muscle contractions with at least 5 out of 10 of the contractions having an amplitude of more than 50 μV peak-to-peak in the relaxed abductor pollicis brevis (23).

After the RMT was obtained, the protocol of the rTMS sessions was determined. Patients were treated with active 20-Hz rTMS over the left dorsolateral prefrontal cortex (DLPFC). rTMS pulses were delivered using a stimulator (YRD, Wuhan, China) with a figure-of-eight focal coil. The coil was placed tangentially to the scalp over the left DLPFC (F3 position according to the International 10–20 EEG System). One session of the rTMS procedure consisted of 2,000 pulses (20 Hz, trains of 20 pulse over 1 s, at intervals of 20 s) at an intensity of 100% RMT. All patients received an rTMS course consisting of 20 sessions five times a week over four consecutive weeks. To ensure uniformity in rTMS sessions as much as possible, the location and orientation of stimulation in all patients were performed by the same skilled physical therapist.

### Behavioral Assessment

The Coma Recovery Scale-Revised (CRS-R) was administered by the same experienced physician before the first and after the last session of rTMS. Responders were defined as patients who had a significant increase in their CRS-R score from baseline to the last session of rTMS (CRS-R score increased by at least 3).

### EEG Data Recording and Processing

Resting-state EEG was recorded from a stable signal for no < 10 min before and immediately after the first session of rTMS. EEG data were acquired through the EEG System with a TMS-compatible amplifier (NuerOne, Bittium Bio-Signals Ltd, Finland) and an EEG Ag/AgCl cap with 19 channels (GREENTEK, China). According to the International 10–20 EEG system, 19 electrodes consisting of Fp1, Fp2, F7, F8, F3, F4, C3, C4, P3, P4, T3, T4, T5, T6, O1, O2, Fz, Cz, Pz, and FCz electrodes were set as an online reference. The impedance of all electrodes was maintained below 5 kΩ, and the sampling rate was 1,000 Hz. Patients were lying in bed, were blindfolded in a quiet room, and wore earplugs to reduce the noise disturbance induced by rTMS. During the rTMS session, an EEG cap was kept on the head, which recorded the signal immediately after the end of the session.

EEG data were processed offline using EEGLAB14.0 running in MATLAB 2018 (Mathworks, Natick, MA, USA). Data were referenced to average reference and band-pass filtered between 0.5 and 48 Hz. Drifting and non-obvious EEG signals were rejected based on visual inspections, and subsequently, an independent component analysis was carried out to remove artifact components, including eye blinks and muscle contractions. For further analysis, 120 s of clear data were extracted. Fast Fourier transform (2 s Hamming) was applied to estimate the EEG power at different frequency bands. Absolute power was obtained for the delta (0.5–4 Hz), theta (4–8 Hz), alpha (8–13 Hz), beta (13–30 Hz), and gamma (30–40 Hz) bands of the whole brain and all electrodes (24). The relative power calculated from absolute power was used to measure the EEG after-effects induced by rTMS at the delta, theta, alpha, beta, and gamma bands.

### Statistical Analyses

A paired *t*-test or Wilcoxon test was performed to analyze the differences in quantitative indicators by comparing the relative power of pre-rTMS with post-rTMS. The independent sample *t*-test and Mann-Whitney *U* test were used to compare the differences in quantitative indicators between groups (e.g., length of time the patient had DOC). The Fisher's exact test was applied to examine the differences in gender, surgery and etiology. Receiver operating characteristic (ROC) curves were used to estimate prediction algorithms to discriminate between DOC patients with improved consciousness and DOC patients who did not have improved consciousness. An area under the ROC curve >0.75 was considered consistent with a good discrimination ability (25). The best cutoff value of the score that predicts the primary endpoint was determined from the ROC curve. All data analyses and graphic designs were made in SPSS version 20, OriginPro 9.1 and MATLAB 2018.

## Results

### Clinical Characteristics Between Groups

Twenty-five patients with DOC were enrolled in this study. Of these 25 patients, 10 patients (responders) had improved consciousness and 15 patients (non-responders) did not have improved consciousness. There were no statistical differences in age, gender, CRS-baseline, and the ratio of patients who previously had surgery between groups. The length of time of patients with DOC was also not significantly different between non-responders and responders. No significant difference was found in the arousal efficacy induced by rTMS between TBI and stroke patients. In comparison to stroke and TBI patients, DOC patients, as a result of HIE, were less likely to have improved consciousness during or after the rTMS protocol as none of the six HIE patients had improved consciousness. The details are summarized in Table 1.

**Table 1:** Clinical characteristics.

**Variable**	**Responders**	**Non-responders**
Number of patients	10	15
Age (years)	52.10 ± 11.66	45.60 ± 11.62
Range of disease duration	3 ~ 9	3 ~ 10
Disease duration (months)	5.00 ± 1.49	5.20 ± 2.39
**Gender (*****n*****)**		
Male	3	13
Female	7	2
**Surgery (*****n*****)**		
Yes	4	6
No	6	9
^*^Etiology (***n*****)**		
TBI	5	4
Stroke	5	5
HIE	0	6
**CRS-R**		
Baseline	5.15 ± 1.55	5.0 ± 1.41
^*^Last session	12.60 ± 1.96	5.47 ± 1.41

TBI, Traumatic brain injury; HIE, hypoxic-ischemic encephalopathy;

* *P < 0.05*.

### EEG After-Effects Induced by rTMS

**Figure 1:**
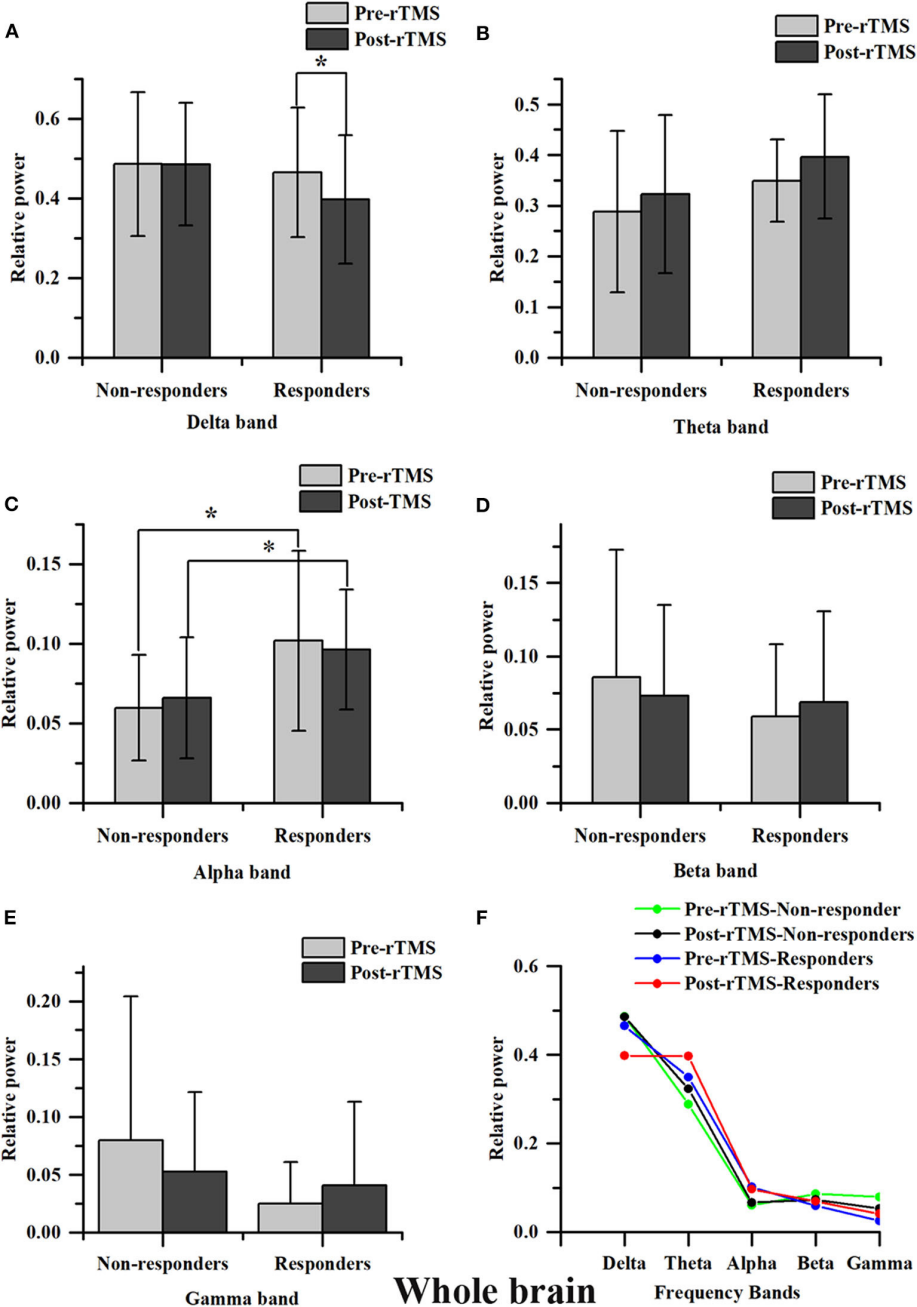
Relative power in the whole brain. A statistical difference within groups was only found in the delta relative power in responders **(A)**. rTMS decreased the delta relative power. A difference between groups was only found in the alpha relative power **(C)**. The alpha relative power in responders is significantly higher compared to that in non-responders, regardless of it being measured before or after rTMS. However, the difference in the alpha relative power before and immediately after rTMS between groups was not statistically significant ^*^P < 0.05. No significant difference was found in theta, beta and gamma bands **(B,D,E)**. Relative power changes in five frequency bands **(F)**.

**Figure 2:**
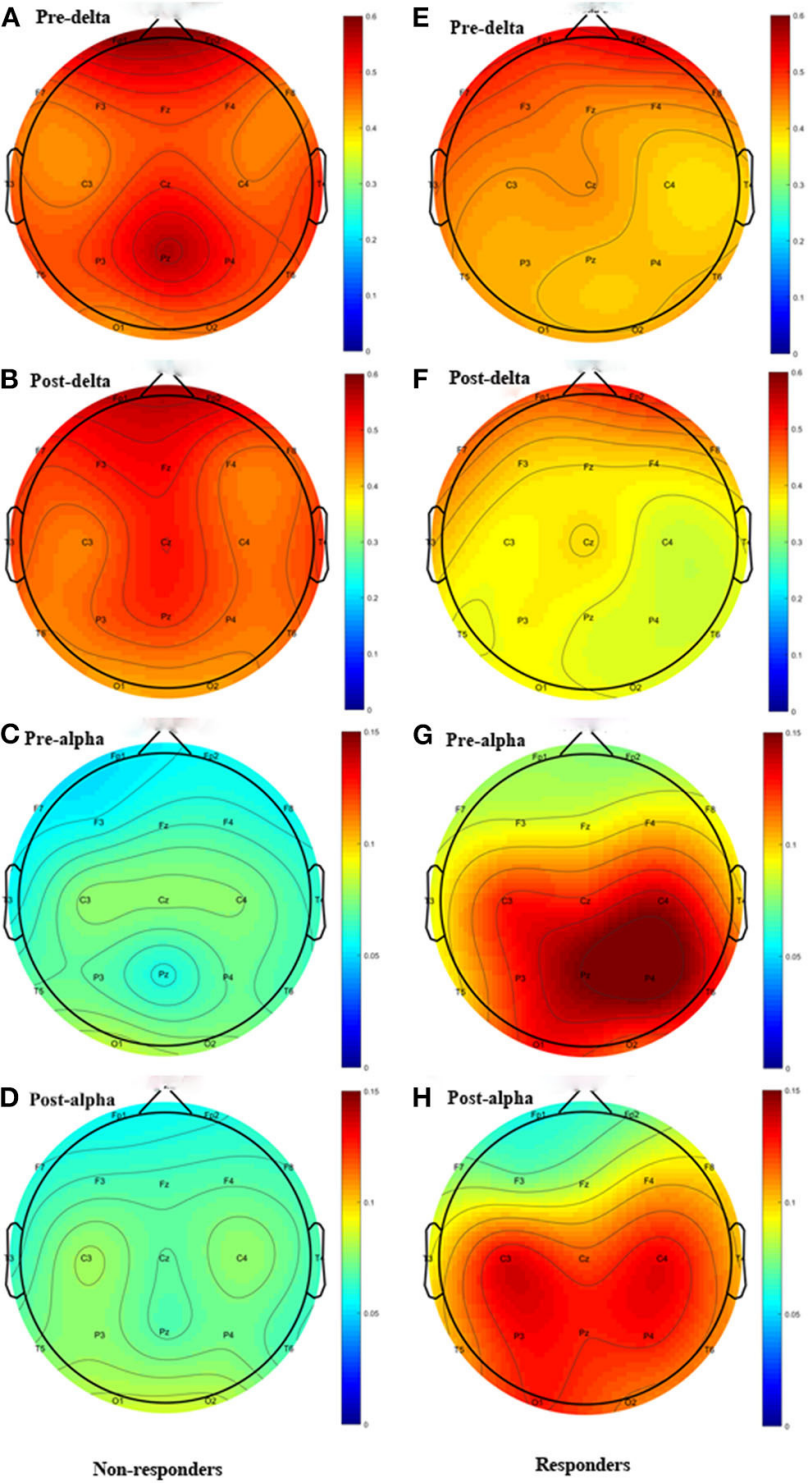
Topographic distribution Figure of relative power. Left column: non-responders; right column: responders. Only the delta and alpha bands are plotted. The highly preserved alpha power in responders is a noticeable feature. **(A)** Delta power of pre-rTMS in non-responders. **(B)** Delta power of post-rTMS in non-responders. **(C)** Alpha power of pre-rTMS in non-responders. **(D)** Alpha power of post-rTMS in non-responders. **(E)** Delta power of pre-rTMS in responders. **(F)** Delta power of post-rTMS in responders. **(G)** Alpha power of pre-rTMS in responders. **(H)** Alpha power of post-rTMS in responders.

Except the relative power of the alpha band, we did not find any significant differences in the relative power of the delta, theta, beta or gamma bands between groups before and immediately after rTMS. Compared with the non-responders, the relative power of the alpha band was significantly higher in the responders (*t* = 2.350, *P* = 0.028), and more alpha band power was preserved in the parietal area (P3 & P4) before rTMS (Figures 1C, 2C,D,G,H). After one session of rTMS, the relative power of the alpha band remained higher in responders compared with non-responders (Figure 1C). However, no statistically significant differences were observed in the relative alpha power before and immediately after rTMS in either group, which demonstrated that there were no rTMS after-effects on the alpha band. In addition, paired *t*-tests and Wilcoxon tests showed that there were no significant differences in the rTMS after-effects on the five frequency bands in the non-responders (Figure 1). However, rTMS induced a significant decrease in the delta relative power in the responders compared with prior to rTMS (*t* = 2.240, *P* = 0.042) (Figure 1A). More specifically, the delta relative power was decreased at the rTMS stimulation site (F3). There were no other significant differences found in the other electrodes between and within groups. The supplementary material shows the details of the different electrodes for each group (https://data.mendeley.com/datasets/vgbghtzvt2/4).

In summary, there was more alpha power in the parietal area before rTMS and rTMS induced a decrease in the relative power at the delta band in responders compared to non-responders, which suggested that these are salient features in responders.

### Outcome Prediction

**Figure 3:**
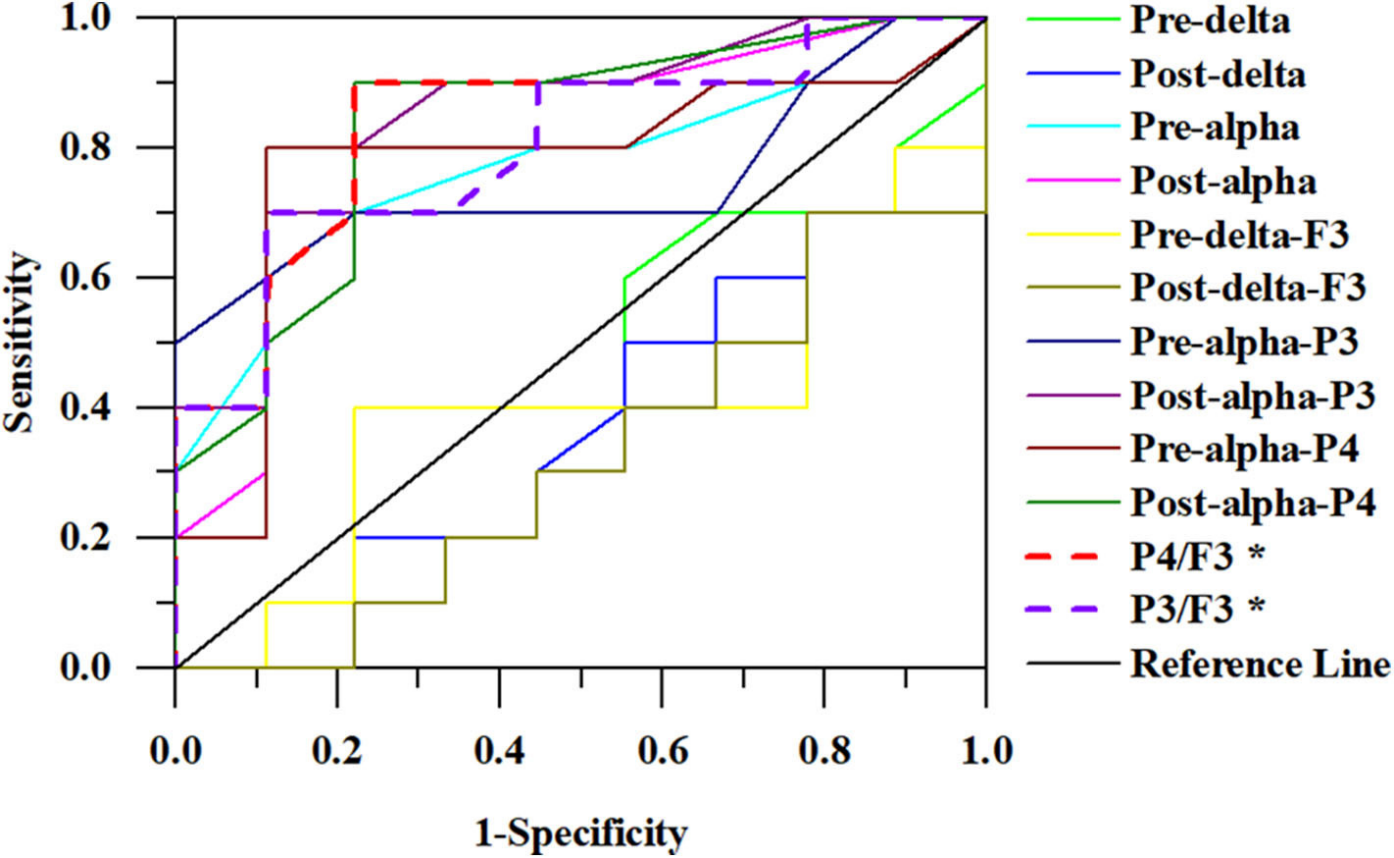
ROC curves for responders. The ratio of alpha relative power in P4 to delta relative power in F3 has the largest area (0.839).

Some algorithms were proposed as classifiers to identify the responders from the non-responders based on the above results and these were evaluated by the ROC curve. The areas under the ROC curve were as follows: 0.394 and 0.361 for the delta relative powers before and after rTMS, respectively; and 0.783 and 0.811 for the alpha relative powers before and after rTMS, respectively. In different electrodes, the areas under the ROC curve were as follows: 0.400 and 0.322 for the delta relative power before and after rTMS in F3, respectively; 0.750 and 0.833 for the alpha relative power before and after rTMS in P3, respectively; and 0.778 and 0.833 in the alpha relative power before and after rTMS in P4, respectively. We also calculated the ratio of the alpha relative power before rTMS in P3 or P4 to the delta relative power after rTMS in F3. The areas were 0.839 and 0.806 for P4/F3 and P3/F3, respectively. Overall, the algorithm calculated from the alpha relative power in the parietal lobe and the delta relative power in the frontal lobe had the highest discriminant accuracy. Table 2 and Figure 3 present the details of the ROC area and statistics.

**Table 2:** Area under the curve.

	**Area**	**Std. Error**	**Asymptotic Sig**	**Asymptotic 95% confidence interval**	
				**Lower bound**	**Upper bound**
Pre-delta	0.394	0.133	0.438	0.134	0.655
Post-delta	0.361	0.130	0.307	0.106	0.616
Pre-alpha	0.783	0.112	0.037	0.564	1.003
Post-alpha	0.811	0.104	0.022	0.608	1.014
Pre-delta-F3	0.400	0.139	0.462	0.128	0.672
Post-delta-F3	0.322	0.125	0.191	0.078	0.567
Pre-alpha-P3	0.750	0.124	0.066	0.508	0.992
Post-alpha-P3	0.833	0.097	0.014	0.644	1.000
Pre-alpha-P4	0.778	0.120	0.041	0.543	1.013
Post-alpha-P4	0.833	0.097	0.014	0.644	1.023
**P4/F3**	**0.839**	**0.097**	**0.013**	**0.648**	**1.029**
P3/F3	0.806	0.104	0.025	0.602	1.009

*Bold values indicates the highest area*.

## Discussion

In this study, EEG after-effects in DOC patients induced by one session of rTMS were used for the first time to identify responders that obtained consciousness improvements. The results from this study show that DOC patients with more preserved alpha power in the parietal lobe and those that had significant reductions of delta power induced by rTMS are more likely to regain consciousness. In addition, we proposed a prediction algorithm that may be promising in discriminating responders from non-responders by using the ratio of the alpha power before rTMS in the parietal lobe to the delta power after rTMS in the left DLPFC.

Even though only six HIE patients were enrolled in this study, our findings suggested that there were no effects of rTMS treatment in DOC patients whose DOC was caused by HIE. Cardiac arrest (CA) is a leading cause of HIE and despite significant improvements in both resuscitation for CA and post-resuscitation care, the mortality rate remains high. According to recent data from the American Heart Association (26), the survival rate for the approximately 356,000 cases of CA that occur outside the hospital is 12% and the survival rate for the approximately 20,900 cases of CA that occur in the hospital is 25% in the United States. Strikingly, only 8% of those that survive CA have good neurological outcomes, which are closely related to the HIE etiopathogenesis (26). In addition, the brain is sensitive to ischemia and hypoxia and can be damaged as a direct result of loss of blood flow as well as reperfusion after successful resuscitation (27). Furthermore, neurological disorders that occur after CA are associated with the vulnerability of the cortex, thalamus, cerebellum and brainstem (28). The vast majority (80–90%) of patients successfully resuscitated from CA present in a coma or an altered level of consciousness, which are both caused by the widespread nature of the brain jury after CA (29, 30). Arousal from a coma is thought to be the best predictor for the post-CA outcome with quicker arousal strongly indicating a better long-term outcome. Hence, once diagnosed with UWS, HIE patients have a scarce possibility to recover consciousness regardless of the therapeutic intervention used, which is supported by a study that showed that neurological recovery is rare in CA patients discharged in a coma during long-term follow up. In a previous study, it was also shown that the treatment outcome in the hospital is almost the ultimate outcome (31). Therefore, based on the results, we disagree with the potential benefits of rTMS in consciousness of HIE patients.

**Parietal and occipital alpha-band power may be a predictor of recovery in UWS patients, and the higher the power, the higher the chance to recover consciousness** (32)**. Our findings led to a similar conclusion that patients with more preserved alpha power in the parietal area were more likely to regain improved consciousness**, which indicated that alpha power detected by EEG can provide valid information to reflect the consciousness state. Alpha-band activity is the dominant oscillation in the awake human brain and constitutes an important neural substrate for cognition and conscious awareness (33). In addition, alpha-band activity has been shown to increase with the load of working memory during working memory maintenance, presumably in order to facilitate working memory retention by preventing interfering inputs (34). Many neurological disorders correlate with abnormity in alpha-band oscillations. For example, major depressive disorder and cognition impairment correlate with alpha-band asymmetry and low alpha-band power, respectively (35, 36). In addition, several reports have shown that cortical alpha rhythms are abnormal in persistent vegetative states and are predictive of the outcome at 3-month follow ups (32, 37). Generally, the VS/UWS patients show increased delta power, but decreased alpha power when compared with MCS patients (38). Therefore, the data presented in previous studies provide a framework to enable the relationship between improved consciousness and more preserved alpha power to be understood in the context of consciousness recovery in DOC patients. Furthermore, our study highlights the role of higher relative alpha power in the parietal area in differentiating the responders from the non-responders in DOC patients. However, a higher alpha power only indicates a better brain state and is not effective in distinguishing benefits in conscious awareness for DOC patients. Several lines of evidence show that improvements in the EEG dominant frequency (from the theta to the alpha band or from the delta to the theta band), reappearance of EEG reactivity and amplitude-frequency-reactivity score increase the ability to discriminate between the patients with improved consciousness from those without consciousness improvement (39, 40). Unfortunately, after one session of rTMS treatment our findings did not show a significant change in the alpha band oscillation in DOC patients regardless of whether patients were responders or non-responders.

Changes in EEGs reflect a remodeling of the electrical brain activity and organization due to neuroplastic adaptations that attempt to restore brain connections and functions after severe brain injury. A systematic review and meta-analysis show that oscillatory EEG responses were the only significant predictor for consciousness improvement (41). An important finding of our study was that one session of rTMS induced a significant reduction of delta relative power in responders, particularly at the stimulation site, which was the left DLPFC. However, there were no rTMS induced after-effects observed in the non-responders. Delta oscillations are known to be highly characteristic of brain pathophysiology, and delta power is strongly correlated with admission NIHSS scores in patients with acute stroke (42). In addition, our study also demonstrated a remarkably high delta power in most DOC patients, except for some DOC cases because of HIE. Furthermore, there was no statistical difference of the delta power in DOC patients regardless of their final outcome before rTMS intervention. Collectively, the reduction of the delta band being induced by rTMS is a distinctive signature in responders.

Consciousness involves widespread activity in the brain, but this does not necessarily imply homogenous activity throughout the brain. During consciousness, brain activity is distributed throughout diffuse regions of the brain, in contrast to unconscious states where activity supposedly remains localized as a result of a lack of connectivity between brain regions (43). Consciousness is dependent on the brainstem and thalamus for arousal, and basic cognition is supported by recurrent electrical activity between the cortex and the thalamus. In addition, for consciousness, some working memory must, at least fleetingly, be present for awareness to occur (6). EEG responses can represent information integration across different brain areas. Therefore, delta power being decreased by rTMS may indicate a preserved connectivity between different brain areas, which might be the precondition of regained consciousness. These ideas are supported by similar findings that show that frontal stimulation may trigger and/or modify the activation of cortical oscillators (44). A single high-frequency rTMS session can modulate the cortical excitatory/inhibitory dynamics, thereby allowing to reach the threshold necessary to recruit some dormant circuits within the frontoparietal networks, and may improve consciousness via restoring the connectivity within several cortical areas in some patients with UWS (19).

Taken together, our findings suggest that more preserved alpha power in the parietal lobe and the modification of the delta band at the stimulation site are distinct features of DOC patients who have improved consciousness. Accordingly, we proposed an algorithm calculated from the alpha power in the parietal lobe and the delta power in the frontal lobe that may serve as a prediction algorithm, which shows promise in predict consciousness improvement in DOC patients. Previous studies have indicated that consciousness as a whole consists of internal and external consciousness (45). In this study, internal consciousness is associated with the default mode network (DMN) (46) and external consciousness is associated with the frontoparietal cortex network (46). Importantly, it is generally acknowledged that rTMS can entrain brain oscillatory activity in a frequency-dependent manner (47), improve brain electrical activities in stroke patients with consciousness disorders, and ultimately promote the recovery of consciousness (48). Therefore, our prediction algorithm is consistent with the neuromodulation by rTMS and the functional brain reconstruction in the recovery of consciousness. Notwithstanding the lack of consensus on the therapeutic efficacy of rTMS in the improvement of consciousness, we believe that rTMS can promote the recovery of consciousness or at least accelerate the recovery rate in responders, who can now be distinguished using our prediction algorithm.

Our study has several limitations. Firstly, the ideal experimental design to propose a prediction algorithm was not ideal because of the relatively small sample size. Secondly, this study is based on the hypothesis that rTMS can improve consciousness in DOC patients. A randomized, sham-controlled study is needed to investigate the efficacy of rTMS in responders who are screened according to the prediction algorithm. Thirdly, the DOC patients in this study were from different etiologies, which may affect the results of this study. For example, in our study, there was no convincing efficacy of rTMS in HIE patients. Nevertheless, we still propose a prediction algorithm that considers different etiologies in DOC patients. Interestingly, the prediction algorithm described here can be used to rule out these HIE patients. Last but not least, the parameter of the rTMS protocol (e.g., the frequency, intensity, pulses, stimulation site, etc.) may also be considered in investigating the EEG after-effects. However, due to the limited sample size, this was not possible in the current study.

In conclusion, this study provides novel insight into DOC patients that responders are characterized by more preserved alpha power in the parietal lobe and a significant reduction of the delta band in the frontal lobe induced by rTMS. Additionally, we propose a prediction algorithm that may be a promising tool to identify responders from non-responders in DOC patients. Future studies to evaluate the algorithm efficacy are warranted.

## Data Availability Statement

The raw data supporting the conclusions of this article will be made available by the authors, without undue reservation.

## Ethics Statement

The studies involving human participants were reviewed and approved by Medical Ethics Committee, Nanfang Hospital, Southern Medical Universtiy. The patients/participants provided their written informed consent to participate in this study.

## Author's Note

In the delta relative power in responders (A). rTMS decreased the delta relative power. The difference between groups was only found in alpha relative power (C). The alpha relative power in responders is significantly higher than in non-responders, regardless of it being measured before or after rTMS. However, the difference of alpha relative power before and immediately after rTMS between groups was not statistically significant ^*^P < 0.05 responders. Only the delta and alpha bands were plotted. The highly preserved alpha power in responders is a noticeable feature.

## Author Contributions

RH collected the data and drafted the manuscript. JF developed the rTMS protocol. YZ, JF, and HW were involved in the recruitment of the patients. JM guided this study and revised the manuscript. All authors contributed to the article and approved the submitted version.

## Conflict of Interest

The authors declare that the research was conducted in the absence of any commercial or financial relationships that could be construed as a potential conflict of interest.
